# Clinic of Jefferson Medical College

**Published:** 1852-09

**Authors:** J. Aitken Meigs


### CLINICAL REPORTS.

*Clinic of Jefferson Medical College. Service of* PROFESSOR DUNGLISON. *February*, 1852.

(Reported by J. AITKEN MEIGS, M. D.)

We to-day direct your attention, in the first place, to a case of intermittent fever.

W. J. R., æt. 18, comes to us laboring under that particular form or type of this disease, known as the quotidian—the paroxysm occurring daily about 10 o'clock in the morning. He has been suffering from the affection about two weeks, and although recourse has been had to the great antiperiodic,—quinia,—it appears to have exerted but little of its controlling influence on his disease. One reason for this may be his continued residence in a malarious district. He lives in the south-western limits of the city, —near the Schuylkill river,—a locality in which, during the vernal and autumnal seasons, intermittent and remittent affections are still very common; though, in consequence of the improvements lately made in this section of the city, by no means as prevalent as formerly. The banks of the river, throughout their entire length, were at one time so rife with the malarious principle,—whatever that may be,—as utterly to preclude all idea of a protracted residence in the vicinity, and many of the country mansions along the river, erected and owned by citizens of this town, were rendered, thereby, entirely untenable during a great part of the year.

The autumn generally presents us with a larger number of intermittents than the other seasons of the year; and I am inclined to consider, that the present case, though occurring, as you see, in the winter season (February), really originated in the previous autumn, and has been elicited, or made itself apparent at this late period, in consequence of accidental circumstances. Indeed, some writers are of opinion, that in all these cases, even in those occurring in the spring, the primary impression is made during the autumnal months. Exposure to the malarious cause, during the fall, may be insufficient to produce immediately an open attack of the disease; but the morbid impression after remaining in the system dormant, as it were, through the winter, is suddenly brought into active operation by the slightest excitation, as exposure to cold, heat, unusual fatigue, mental inquietude, &c., and the febrile paroxysm follows.

I have long maintained the opinion, that it would not be improper to class intermittent fever among the neuroses. It certainly displays, in its course, very marked neuropathic phenomena. The miasmatic impression falls primarily upon the nervous system, with greater or less intensity, producing the cold or congestive stage, as a result of the extreme nervous depression or sedation. The system soon begins to react; the cutaneous capillaries gradually become filled, to the immediate relief of the congested viscera; and after a few hours of alternate flushing and chilliness, the hot or febrile stage—the stage of expansion—is fully established. Finally, the paroxysm resolves itself by a copious diaphoresis, and during the ensuing apyrexia or intermission, the patient, with the exception of a certain degree of debility, may enjoy his usual health. If the disease be not now checked, sooner or later, according to the nature of the type, a second attack occurs; and then a third; and so on until an arrestation is produced either spontaneously or by therapeutical intervention.

The intervals between the paroxysms are of the same duration; in other words, the disease is periodical in its character. Now, although our ignorance of the nature of the laws of periodicity is very profound, yet as we see these laws manifested in remittent and hectic fevers, as well as in the disease under question,—and also in certain functions of the healthy economy, as the menstrual act,—sufficient is known to render it highly probable, that this periodicity is closely connected with the functional manifestations of the nervous system. This supposition of the nervous character of intermittent fever, is further substantiated by the fact, that the paroxysm may oftentimes be arrested by the sudden production of a moral emotion. The cold stage has been completely checked, for the time being, by fright, or by the receipt of highly joyous or very painful intelligence. Mesmerism,—the power of which depends altogether upon the impressibility of the nervous system,—will sometimes produce the same desirable effect. I recollect an anecdote, illustrative of this, told me by Ex-president Madison. Mr. M. was travelling in company with Lafayette, who had been inducted into the mesmeric mysteries by the celebrated Loutherberg, a pupil of the far-famed Mesmer, when John, the servant of the former, was seized with a paroxysm of intermittent. Wishing to test the mesmeric powers of his distinguished friend, Mr. Madison recommended a trial of the art. Lafayette complied with the advice. The paroxysm was commencing, when Lafayette stared John in the face, seating himself in front of him, and holding him firmly by the hands. In a few minutes, John declared that he felt better; and by a continuance of the process, the paroxysm was entirely prevented.

Again;—our potent therapeutical agents, tonics and antiperiodics, employed in the disease, strongly bear out the opinion of its neuropathic character. They all prominently affect the nervous system, and are valuable in proportion to the degree of impression which they are calculated to make on the nerves of gustation, and on those distributed to the alimentary canal. The correctness of this view is proved by the fact, that the salts of quinia are much more efficient in solution, than in pill,—according to one observer, one third more. Furthermore, it not unfrequently happens, that when the sulphate of quinia fails to arrest the paroxysm, opium, in the dose of gr. ij., or an equivalent of any of its preparations, given at the moment when the lividity and contraction of the skin begin to announce the attack, will often effectually arrest it, in consequence of the narcotic influence, which it exerts, manifestly on the nervous system.

The propriety, then, of ranking periodical fevers with neurotic affections is founded upon the threefold consideration of the modus operandi of the cause of the disease, its periodical character, and the effects of the agents employed for its removal.

In the case before us, there is, as you can readily ascertain by percussion and inspection, some enlargement of the spleen, a common, and by some thought a universal, accompaniment of this form of fever. This increased size of the spleen is attributed with much propriety to the undue accumulation of the circulatory fluid in the vascular apparatus of the organ. Indeed its tendency to become thus congested during the cold stage is one of the reasons in favor of its acting as a diverticulum to the blood under like circumstances; since, from the great distensibility of its tissue, it is much better able than any other viscus to withstand the pressure incident to the extreme engorgement to which it is, at times, subjected in the disease under consideration. In chronic cases, however, the increase in volume is mainly dependent upon hypertrophy of the substance of the organ, or upon altered nutrition, induced by repeated and long continued engorgements.

In tropical climates, as in India, the splenic affection sometimes assumes an exceedingly dangerous and formidable aspect. The whole system deteriorates, the blood becomes depraved, and hemorrhage from the gastro-enteric mucous surface, or hydropic effusions, ensue. To this distressing condition, the name of "splenic cachexia" has been given by the practitioners of India more especially. Independently of the engorgement of the spleen, the irregularity in the circulation, in the various stages, by disturbing the healthy equilibrium between the absorbents and exhalents, frequently gives rise to œdema—another pathological point of importance, but which is not presented in the case now before you.

As regards the treatment, but little need be said: an ordinary ague is, indeed, so readily managed, that, frequently, the aid of the physician is not sought after. During the first or cold stage of the paroxysm, as the blood manifests a centripetal tendency, producing, at times, great and dangerous congestion of certain vital organs, as the brain, lungs, &c., every attempt should be made to re-establish the sanguineous equilibrium, by inviting an afflux of blood into the peripheral capillaries. Especially should our efforts be so directed, where the nervous sedation is extreme, as well as unduly protracted. The warm bath at this period is highly beneficial, in consequence of favoring the speedy supervention of the stage of expansion or reaction, by the determination it produces towards the cutaneous surface.

In the hot stage, the cooling system must be adopted throughout; whilst but little attention, excepting to the comfort of the patient, is needed in the sweating stage.

During the intermission, as you are aware, our principal efforts for the eradication of the disease are to be made, and for this purpose the salts of quinia, as the sulphate, or some of the preparations of cinchona, constitute our main reliance. Having obtained a clear mucous surface by the exhibition of an emetic or cathartic, if either or both of these agents seem to be indicated, we administer the antiperiodic in the dose of five grains about one hour before the attack. In half an hour, the dose is repeated, and, again, about the time the paroxysm is expected. I prefer this mode of administering the remedy to that adopted by many physicians, of distributing it over the entire apyrexia.

Ten grains given in solution I have found succeed in arresting most of the intermittents that have fallen under my care in the first instance, and where the paroxysms have not often recurred—one dose of five grains given an hour, and the other dose half an hour, before the paroxysm, as in the following mixture:

R. Quiniæ sulphat., gr. xv.
Acid. sulphur. dil. gtt. xxv.
Aquæ f. ℥iij. M.

Dose, one-third.

The great object to be kept in view is to make a new impression on the nervous system, so decided as to break in upon the morbid chain of associated actions, formed under the malarious influence.

The salts of quinia were, at one time, considered to be excitant, and they were always interdicted during the hot stage, notwithstanding it had been an old observation, that when powdered cinchona was given during the hot stage, it did not appear to excite the organic actions, whilst its antiperiodic virtues were not the less manifested. Careful and well directed inquiries have shown, that full doses are decidedly sedative; and hence, instead of apprehension being entertained from their administration where inflammatory action is present, they have even been found useful in the most violent inflammatory diseases—pneumonia, for example, when occurring in malarious regions. It has been a misfortune, that therapeutists have been misled in regard to some of our most valuable drugs, and this at times owing to an improper classification of them according to their presumed operation on the system. Cinchona and the salts of quinia being tonic, it has been thought that they could not fail to be excitant, and yet all admit that there is a manifest difference between healthy tone and unhealthy excitement. The valuable drug, opium, has been withheld in numerous cases, in which its use would have been precious, because it has been improperly classed amongst excitants. It is true, that in a small dose it belongs to that division of therapeutical agents, but in a large dose it is unequivocally sedative, and, hence, admirably adapted for cases in which inflammatory action may have to be combated.

It occasionally happens, perhaps from peculiar idiosyncrasy of the patient, that quinia has not its wonted influence. In such cases, much good may be anticipated from a resort to the arsenical preparations, of which the liquor potassæ arsenitis, of the U. S. Pharmacopœia, is generally chosen. In other instances, pulverized cinchona is productive of much benefit, although its active principle may have had little or no effect on the disease. In these cases the ligneous matter of the bark, in all probability, comes in aid of the alkaloid, by the excitant impression it makes on the nerves of the alimentary mucous membrane. In good cinchona, we have, besides, all the febrifuge principles which may act more beneficially than the isolated quinia.

The sulphate of quinia, though judiciously administered in the present case, has, I said, been productive of little or no good. We must seek, therefore, to impress the nervous system in some other manner. With this object in view, then, the the patient will be directed to take, at about seven o'clock in the morning, an emetic consisting of pulvis ipecacuanhæ gr. xv., to be repeated in a short time if the first dose fails to act promptly and vigorously. Half an hour or an hour after the cessation of the emesis, pulvis cinchonæ is to be exhibited in the dose of grs. xx. every half hour, up to the time of the expected attack. This plan is to be repeated three mornings in succession. By such procedure, we shall be enabled to exert a three-fold action upon the nervous system—through the emetic, the alkaloid, and the woody matter of the bark.

The result of this treatment you will have an opportunity of witnessing in a few days.

[The patient did return, and reported himself cured.]

The class will have observed, that many of the cases of intermittent have been cured by the pulvis cinchonæ administered in this manner; and I have had recourse to the antiperiodic in this form, in consequence of the sulphate of quinia being so constantly prescribed, that you have but little opportunity for witnessing the action of the cinchona itself; which, in eleemosynary institutions like our own, has the advantage, that the cure performed by it is at much less cost. I have rarely had occasion to exceed the half drachm doses given at the same time, and under the same circumstances, as the five grain doses of sulphate of quinia before referred to.

